# Cholesterol efflux regulator ABCA1 exerts protective role against high shear stress-induced injury of HBMECs via regulating PI3K/Akt/eNOS signaling

**DOI:** 10.1186/s12868-022-00748-2

**Published:** 2022-11-05

**Authors:** Zhe Li, Jia-Nan Li, Qiang Li, Chun Liu, Lin-Hua Zhou, Qi Zhang, Yi Xu

**Affiliations:** 1 Present Address: Neurovascular Center, Changhai Hospital, Naval Medical University, No. 168 Changhai Rd, Shanghai, 200433 China; 2 Present Address: Department of Cerebrovascular Diseases, Blue Cross Brain Hospital Affiliated to Tongji University, No. 2880 Qixin Road, Shanghai, 201101 China; 3 Department of Neurosurgery, General Hospital of Northern Theater Command, 83 Wenhua Road, Shenyang, Liaoning Province China

**Keywords:** ABCA1, Atherosclerosis, Cholesterol efflux, High shear stress, Human brain microvascular endothelial cells, PI3K/Akt/eNOS pathway

## Abstract

**Background:**

In brain, microvascular endothelial cells are exposed to various forces, including shear stress (SS). However, little is known about the effects of high shear stress (HSS) on human brain microvascular endothelial cells (HBMECs) and the underlying mechanism. The cholesterol efflux regulator ATP-binding cassette subfamily A member 1 (ABCA1) has been demonstrated to exert protective effect on HBMECs. However, whether ABCA1 is involved in the mechanism underneath the effect of HSS on HBMECs remains obscure. In the present study, a series of experiments were performed to better understand the effect of HSS on cellular processes of HBMECs and the possible involvement of ABCA1 and PI3K/Akt/eNOS in the underlying mechanisms.

**Results:**

HBMECs were subjected to physiological SS (PSS) or high SS (HSS). Cell migration was evaluated using Transwell assay. Apoptotic HBMECs were detected by flow cytometry or caspase3/7 activity. IL-1β, IL-6, MCP-1 and TNF-α levels were measured by ELISA. RT-qPCR and western blotting were used for mRNA and protein expression detection, respectively. ROS and NO levels were detected using specific detection kits. Compared to PSS, HBMECs exhibited decreased cell viability and migration and increased cell apoptosis, increased levels of inflammatory cytokines, and improved ROS and NO productions after HSS treatment. Moreover, HSS downregulated ABCA1 but upregulated the cholesterol efflux-related proteins MMP9, AQP4, and CYP46 and activated PI3K/Akt/eNOS pathway. Overexpression of ABCA1 in HBMECS inhibited PI3K/Akt/eNOS pathway and counteracted the deleterious effects of HSS. Contrary effects were observed by ABCA1 silencing. Inhibiting PI3K/Akt/eNOS pathway mimicked ABCA1 effects, suggesting that ABCA1 protects HBMECs from HSS via PI3K/Akt/eNOS signaling.

**Conclusion:**

These results advanced our understanding on the mechanisms of HSS on HBMECs and potentiated ABCA1/PI3K/Akt/eNOS pathway as therapeutic target for cerebrovascular diseases.

**Supplementary Information:**

The online version contains supplementary material available at 10.1186/s12868-022-00748-2.

## Background

Brain microvascular endothelial cells (BMECs) constitute the blood–brain barrier (BBB) which forms a dynamic interface between the blood and the central nervous system (CNS) [1–3]. This highly specialized interface restricts paracellular diffusion of fluids and solutes including chemicals, toxins and drugs from entering the brain [4]. The alteration in the function of BMECs is involved in the pathogenesis of a variety of cerebrovascular diseases such as stroke and neurodegenerative diseases [5]. Ample evidence suggests that shear stress (SS), a hemodynamic force that is imposed on vascular endothelial cells (ECs) by blood flow [6], is an important regulator of vascular diseases [7], and plays fundamental roles in vascular homeostasis [8]. Since main arteries, as well as large straight vessels are subjected to uniform blood flow and often undergo high SS (HSS) [9], the BMECs are also subjected to HSS. However, the effect of HSS on cerebrovascular diseases and the underlying cellular and molecular mechanisms still need to be deeply explored. The current research findings on the effect of HSS on BMECs remain controversial. Some studies have indicated that HSS can restore the function of BMECs following ischemic injury by controlling the PECAM-1-eNOS-NO pathway [10]. It was also demonstrated that HSS regulates the apoptosis of BMECs [5, 10, 11] and this may be driven by the PECAM-1-eNOS-NO pathway [10]. However, another study indicated that HSS deteriorates and induces impairment of the barrier phenotype in brain microvascular endothelium [12]. Thus, in spite of the progress made in this field, the effect and the underlying mechanism of HSS on BMECs has not been clarified, which needs additional in-depth studies.

ABCA1, also called ATP-binding cassette transporter A1, is an important mediator of cholesterol efflux; due to its diverse regulatory functions, it is ubiquitously expressed throughout the body. Disrupted ABCA1 regulation has been reported to perturb cholesterol removal from the cytoplasm of senescent macrophages, leading to thickened and hardened blood vessel, and culminated in pathologic atherogenesis [13]. In addition, early onset of atherosclerosis induced by ABCA1 mutation was reported to cause brain infarction [14]. Hence, we speculated that ABCA1, by regulating cerebral atherogenesis, might exert cerebral-protective effects. Given that ECs (including cerebral ECs) are constantly exposed to blood flow, and that ABCA1 is involved in endothelial functions, we also hypothesized that ABCA1 might be regulated in BMECs in response to different SS profiles.

Neuroinflammatory and neurodegenerative disorders such as Parkinson's and Alzheimer's diseases or multiple sclerosis are characterized by loss of vascular integrity and chronic inflammation [15–19]. The activated BMECs of the BBB of patients with the above disorders are present at sites of inflammation where they produce cytokines, chemokines, and adhesion molecules and express a number of transcription factors and inflammatory markers such as IL-1β, IL-6, MCP-1 and TNF-α [20–22]. However, the effect of HSS on these inflammatory markers is not well elucidated. In addition, though ABCA1 has been reported to inhibit inflammation [23–27], the regulation of inflammatory markers in BMECs under HSS has not been reported so far, which needs an in-depth study.

Phosphoinositide 3-kinases (PI3Ks), also termed phosphatidylinositol 3-kinases, are a family of enzymes participating in multiple cellular functions including cell growth, motility, survival and shear‐mediated platelet activation [28]; the majority of these properties are mediated by activation of Akt (Protein Kinase B) in PI3K/Akt pathway [29]. Nitric oxide (NO), a product of ECs through endothelial NO synthase (eNOS) in the presence of mechanical stimulation [30], has been proposed as an important mediator for maintaining vascular hemostasis through controlling endothelial permeability [31], inducing vasorelaxation [32] and promoting endothelial survival [33]. Taken together, PI3K/Akt/eNOS axis could work in concert to protect cells from potential damage, and their joint protective effect has been also indicated in a report of intestinal injury [34]. Importantly, unavailability of NO has been reported as a characteristic of endothelial dysfunction, and cholesterol levels have been found to be inversely correlated with NO production [35]. Moreover, ABCA1 has been proposed to be regulated by NO in response to HSS [36]. Therefore, the subtle interplay between cholesterol regulator ABCA1 and NO production regulator eNOS (PI3K/Akt/eNOS) is worth discussing. It has been elucidated that HSS might protect endothelial cells from apoptosis [37], a biological process which impairs the endothelium by inducing atheroprone lipid deposition [38]; in addition, SS could enhance the endothelial-nondirectional migration speed [39], as the migration of ECs is also important for restoring endothelium integrity [40]. Taken together, we hypothesized that inhibition of apoptosis and promotion of endothelial migration might be alternative ways by which SS contribute to the maintenance of endothelium integrity. MMP9 is a mediator of cell migration [41], and contributes to the post-insult recovery of stroke [42] and traumatic brain injury [43]. AQP4 is abundantly distributed in the brain and responsible for water exchange across the BBB and maintenance of ion homeostasis; aberrant AQP4 expression was reported to be associated with cerebral edema/ischemia [44, 45]. Importantly, AQP4 deletion was reported to promote post-intracerebral hemorrhage and apoptosis [46]. In hippocampal neurons, CYP46 offers considerable protection against exogenous stimulations by inducing cholesterol loss [47]. Since cholesterol overload is known to be associated with apoptosis [48], CYP46 bears the potential to counter apoptosis by reducing cholesterol level.

Human BMECs (HBMECs) are an established in vitro laboratory material for investigating neuro-inflammation and disease processes in brain [49]. The present study aimed to investigate the role of ABCA1 in HSS-induced functional changes of HBMECs; a series of experiments were performed to better understand how ABCA1 regulates cellular processes under HSS. The regulatory relationship between ABCA1 and PI3K/Akt/eNOS under HSS was also examined in order to shed light on the potential mechanisms underlying the effect of HSS on HBMECs.

## Results

### HSS alters cellular functions and modulates the ABCA1 expression and PI3K/Akt/eNOS signaling in HBMEC

**Fig. 1:**
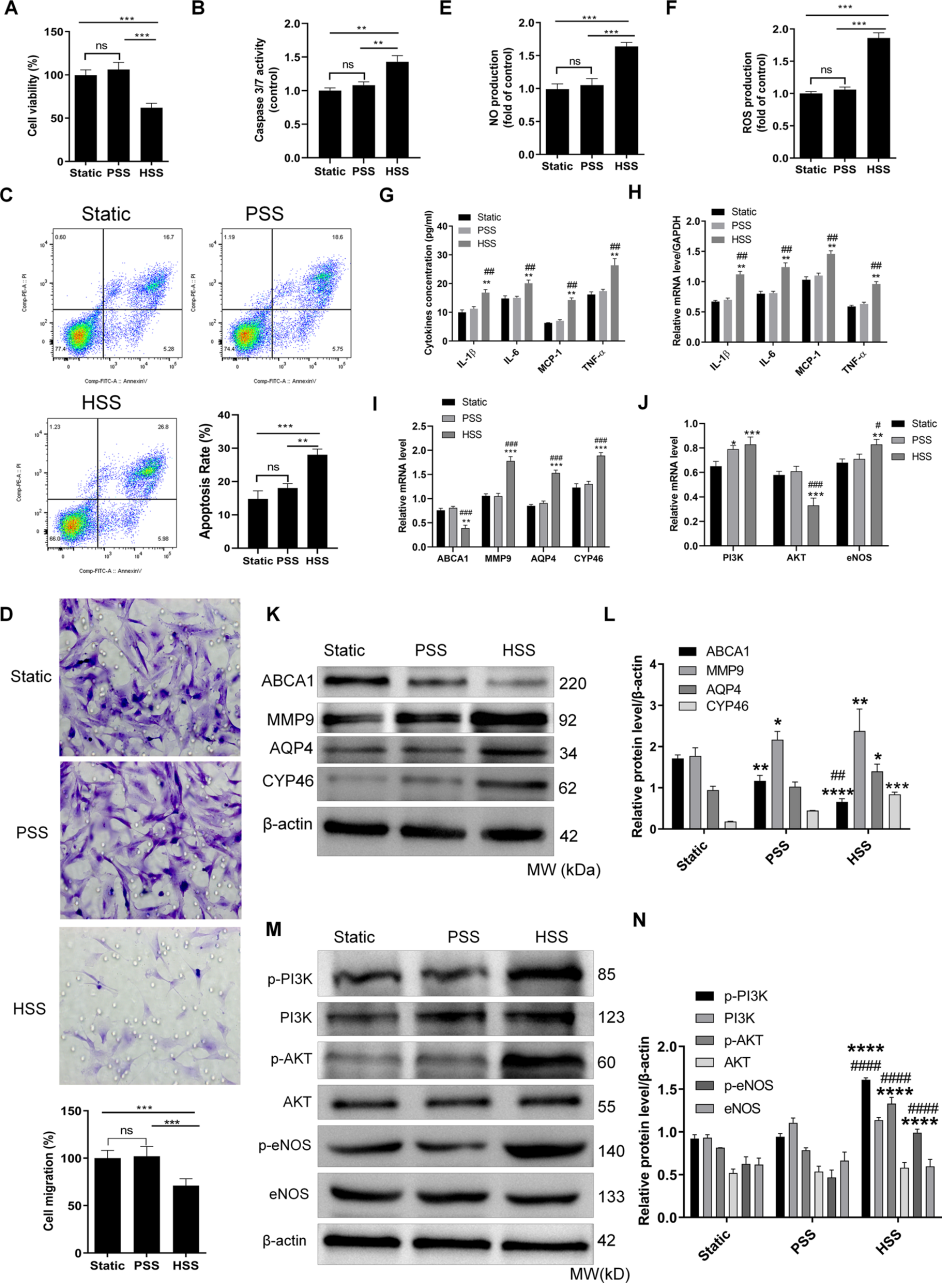
HSS alters cellular functions and modulates the ABCA1 expression and PI3K/Akt/eNOS signaling in HBMEC. **A** Effect of SS on the viability of HBMECs. **B** Effect of SS on the caspase 3/7 activity in HBMECs. **C** Images of flow cytometry analysis of the apoptosis of HBMECs and related numerical quantification. **D** Images of Transwell analysis of the migration of HBMECs and related numerical quantification. **E** NO production in HBMECs. **F** ROS production in HBMECs. **G** ELISA detection of the release of inflammatory cytokines from HBMECs. **H** qRT-PCR detection of the inflammatory cytokines from HBMECs. **I** qRT-PCR detection of ABCA1, MMP9, AQP4, and CYP46 from HBMECs. **J** qRT-PCR detection of PI3K, AKT and eNOS from HBMECs. **K** Western blot images showing the expression of ABCA1, MMP9, AQP4, and CYP46 proteins in different groups. **L** Densitometry analysis of western blot images of ABCA1, MMP9, AQP4, and CYP46. **M** Western blot images showing the expression of PI3K, AKT and eNOS and their phosphorylated forms in different groups. **N** Densitometry analysis of western blot images of PI3K, AKT and eNOS and their phosphorylated forms. ^*^p < 0.05, ^**^p < 0.01, ^***^p < 0.001, ^****^p < 0.0001 versus control or between indicated compared group; ^#^*p* < 0.05, ^##^*p* < 0.01, ^###^*p* < 0.001, ^####^*p* < 0.001 versus PSS group. Full-length blots/gels are presented in Additional file 1: Figures S1 and S2

We investigated the effect of HSS on HBMECs and found that HSS inhibited the viability of HBMECs compared to static or PSS (Fig. 1A). On the contrary, caspase 3/7 activity and flow cytometry detections indicated that HSS induced the apoptosis of HBMECs compared to the static and PSS (Fig. 1B and C). HSS significantly decreased the migration of HBMECs compared to static or PSS (Fig. 1D). Moreover, we found that HSS significantly increased ROS (Fig. 1E) and NO (Fig. 1F) productions in HBMECs relatively to the static and SS groups. Moreover, the levels of cytokines such as IL-1β, IL-6, MCP-1 and TNF-α were increased by HSS at protein (Fig. 1G) and mRNA levels (Fig. 1H). To further identify whether the molecular mechanism underlying the effect of HSS on the HBMECs could be driven via the cholesterol metabolism pathway and the PI3K/Akt/eNOS pathway, qRT-PCR and western blotting experiments were performed. The mRNA expression level of ABCA1 was significantly decreased by HSS while the mRNA expression levels of MMP9, AQP4 and CYP46 were all increased following HSS treatment (Fig. 1I).

Moreover, the mRNA levels of PI3K and eNOS were significantly increased by the treatment with HSS while the level of Akt was significantly decreased compared to the static and PSS groups (Fig. 1J). In the western blotting experiment, the expression of ABCA1 was significantly decreased by the treatment with HSS while the expression levels of MMP9, AQP4 and CYP46 were also boosted by HSS (Fig. 1K and L). Furthermore, the ratios of p-PI3K/PI3K, p-Akt/Akt and p-eNOS/eNOS were all increased after HSS treatment (Fig. 1M and N).

### ABCA1 overexpression counteracts HSS-induced functional alterations in HBMECs

**Fig. 2:**
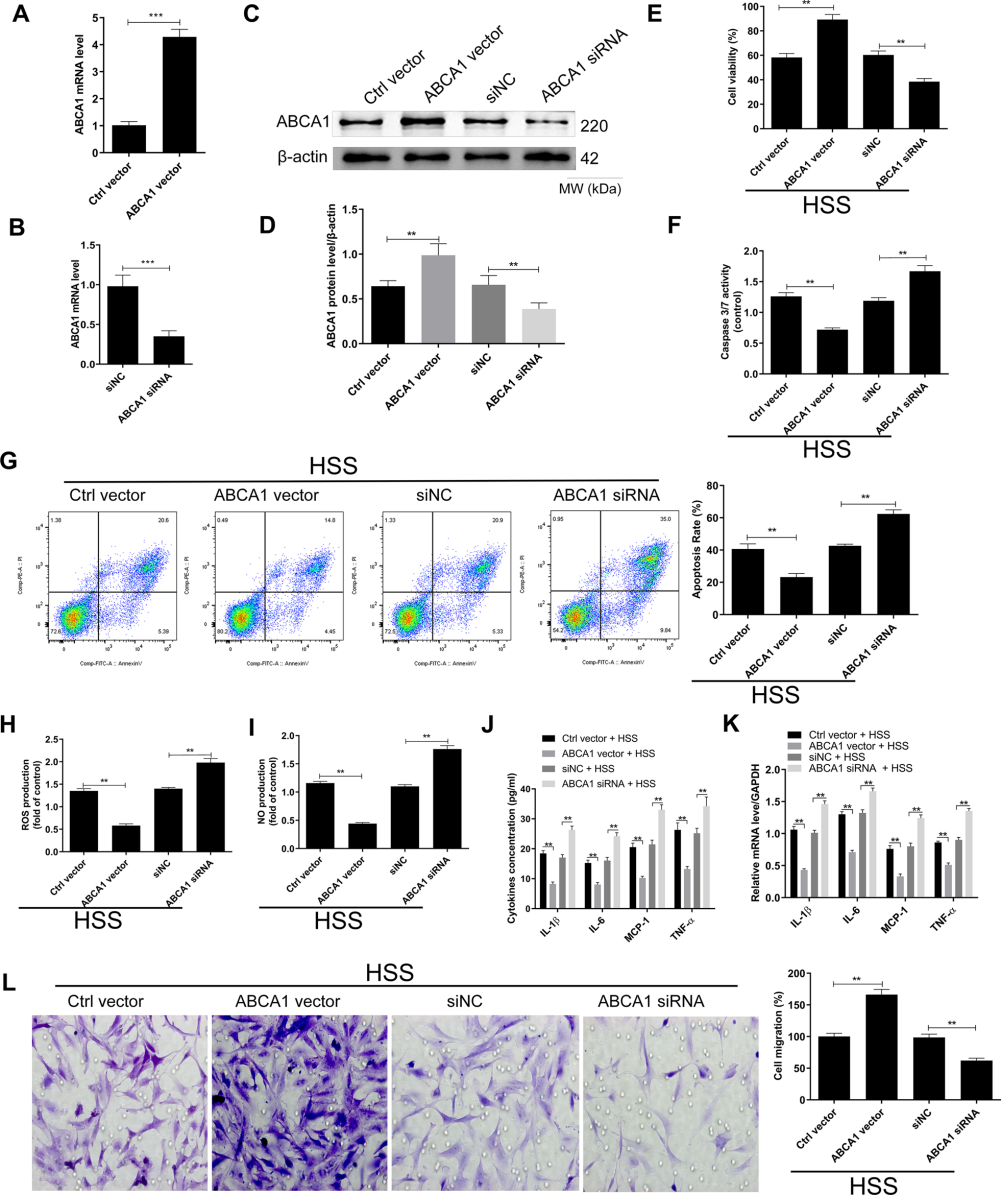
ABCA1 overexpression counteracts HSS-induced functional alterations in HBMECs. **A** qRT-PCR detection of the effect of ABCA1 overexpression vector on ABCA1 mRNA expression. **B** qRT-PCR detection of the effect of ABCA1-siRNA on ABCA1 mRNA expression. **C** Western blot detection of the effect of ABCA1-siRNA and expression vector on ABCA1 mRNA expression. **D** Densitometry analysis of western blot detection of the effect of ABCA1-siRNA and expression vector on ABCA1 protein expression. **E** Effect of ABCA1 on the viability of HBMECs under HSS treatment. **F** Effect of ABCA1 on the caspase 3/7 activity in HBMECs under HSS treatment. **G** Effect of ABCA1 on the apoptosis of HBMECs under HSS treatment as determined by flow cytometry. **H** Effect of ABCA1 on ROS production in HBMECs under HSS treatment. **I** Effect of ABCA1 on NO production in HBMECs under HSS treatment. **J** Effect of ABCA1 on the release of inflammatory cytokines in HBMECs under HSS treatment. **K** Effect of ABCA1 on the mRNA expression levels of inflammatory cytokines in HBMECs under HSS treatment as indicated by qRT-PCR experiment. **L** Effect of ABCA1 on the migration of HBMECs under HSS treatment. ^**^p < 0.01 and ^***^p < 0.001 among the compared groups. Full-length blots/gels are presented in Additional file 1: Figure S3

Based on the foregoing observations, we further investigated whether ABCA1 is a mediator of the mechanism of HSS on HBMECs. First, the overexpression and silencing of ABCA1 was performed. Figure 2A, C and D showed that the mRNA and protein levels of ABCA1 were increased upon transfection with the expression vector. In Addition, the transfection with the ABCA1-siRNA was followed by significant decrease in the expression of ABCA1 (Fig. 2B, C and D). These results indicated high transfection efficiency of the ABCA1 expression vector and ABCA1-siRNA, which implied successful overexpression and suppression of ABCA1. Next, the cells transfected with the ABCA1 vector or ABCA1-siRNA or their related controls were subjected to HSS to investigate the effect of ABCA1 on the function of HBMECs under HSS conditions. As shown in Fig. 2E, the overexpression of ABCA1 was followed by the increase in cell viability of HBMECs while contrary results were obtained by ABCA1 silencing. Moreover, the determination of caspase 3/7 activity and flow cytometry analysis indicated a remarkable decrease of cell apoptosis after ABCA1 overexpression while a contrary effect was recorded after ABCA1 silencing (Fig. 2F and G). Similarly, the ROS production (Fig. 2H), NO production (Fig. 2I), the protein (Fig. 2J) and mRNA (Fig. 2K) levels of IL-1β, IL-6, MCP-1 and TNF-α were all decreased by the overexpression of ABCA1 but increased by the silencing of ABCA1. Moreover, the migration of HBMECs was stimulated by the overexpression of ABCA1 but hindered by the silencing of ABCA1 (Fig. 2L).

### ABCA1 overexpression inhibits the PI3K/Akt/eNOS Pathway under HSS conditions

**Fig. 3:**
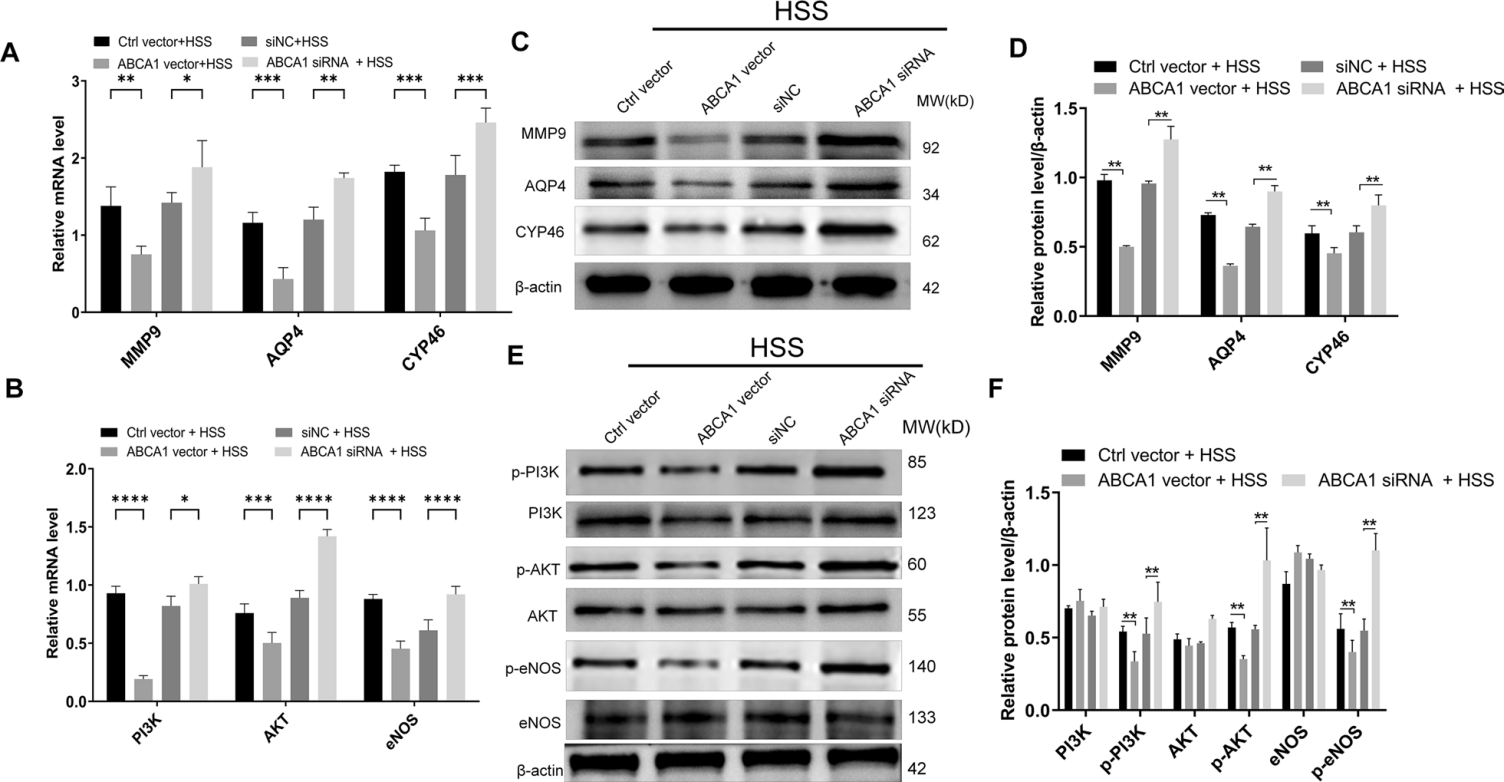
ABCA1 overexpression counteracts HSS-induced gene regulation changes in HBMECs. **A** Effect of ABCA1 overexpression and siRNA on the mRNA levels of MMP9, AQP4 and CYP46 as determined by qRT-PCR. **B** Effect of ABCA1 overexpression and siRNA on the mRNA levels of PI3K, AKT and eNOS as determined by qRT-PCR. **C** Effect of ABCA1 overexpression and siRNA on the protein levels of MMP9, AQP4, CYP46 and their phosphorylated forms as determined by Western blotting. **D** Densitometry analysis of MMP9, AQP4 and CYP46. **E** Effect of ABCA1 overexpression and siRNA on the protein levels of PI3K, AKT and eNOS. **F** Densitometry analysis of PI3K, AKT and eNOS and their phosphorylated forms. ^*^p < 0.05, ^**^p < 0.01, ^***^p < 0.001, ^****^p < 0.0001 among the compared groups. Full-length blots/gels are presented in Additional file 1: Figures S4 and S5

To investigate the potential regulatory effects of ABCA1 on the PI3K/Akt/eNOS pathway, qRT-PCR and western blotting experiments were performed. As shown in Fig. 3A, the mRNA expression levels of MMP9, AQP4, and CYP46 were decreased by the overexpression of ABCA1. Significant effect was recorded on the mRNA expression levels of PI3K and AKT and eNOS (Fig. 3B). Western blotting analysis indicated that the protein expression levels of MMP9, AQP4, and CYP46 were repressed by the overexpression of ABCA1 (Fig. 3C and D). In addition, the expression ratios of p-PI3K/PI3K, p-AKT/AKT and p-eNOS/eNOS were all decreased by the overexpression of ABCA1 (Fig. 3E and F). Inverse results were recorded with ABCA1 silencing (Fig. 3).

### PI3K/Akt/eNOS pathway is involved in HSS-induced alteration in cell migration and apoptosis

**Fig. 4:**
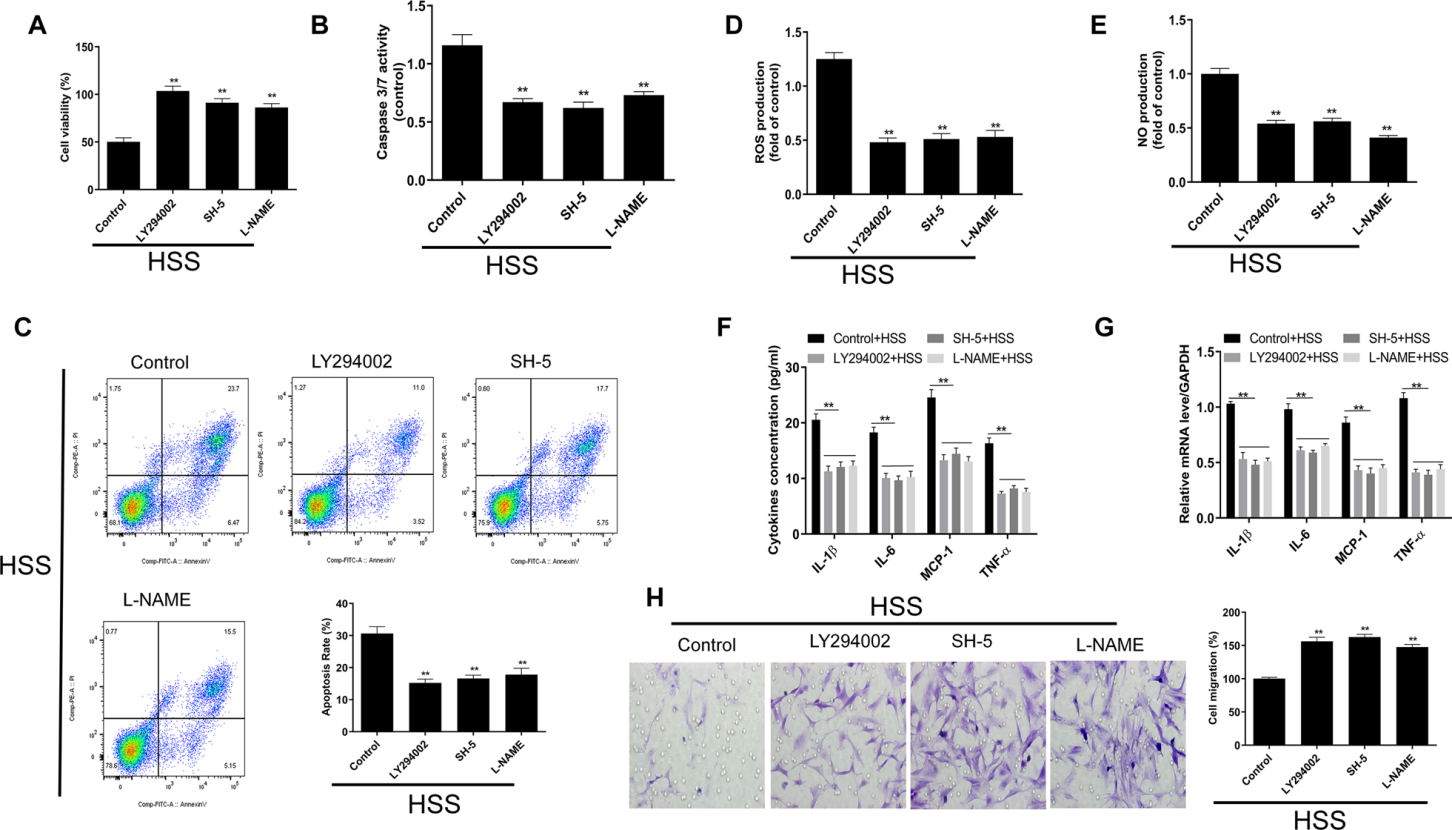
Inhibition of PI3K/Akt/eNOS counteracts HSS-induced functional alterations in HBMECs. **A** Effect of PI3K/Akt/eNOS inhibitors on the viability of HBMECs under HSS treatment. **B** Effect of PI3K/Akt/eNOS inhibitors on the caspase3/7 activity in HBMECs under HSS treatment. **C** Effect of PI3K/Akt/eNOS inhibitors on the apoptosis of HBMECs under HSS treatment as determined by flow cytometry. **D** Effect of PI3K/Akt/eNOS inhibitors on ROS production in HBMECs under HSS treatment. **E** Effect of PI3K/Akt/eNOS inhibitors on NO production in HBMECs under HSS treatment. **F** Effect of PI3K/Akt/eNOS inhibitors on the release of inflammatory cytokines in HBMECs under HSS treatment. **G** Effect of PI3K/Akt/eNOS inhibitors on the mRNA levels of IL-1β, IL-6, MCP-1 and TNF-α as determined by qRT-PCR in HBMECs under HSS treatment. **H** Effect of PI3K/Akt/eNOS inhibitors on the migration of HBMECs under HSS treatment. ^⁎⁎^*p* < 0.01 versus control

To further evaluate the functional roles of PI3K/Akt/eNOS signal transduction cascade under HSS condition, we employed three independent inhibitors, namely PI3K inhibitor (LY294002), AKT inhibitor (SH-5) and eNOS inhibitor (L-NAME), to suppress the activation of PI3K, Akt or eNOS. HBMECS were incubated with 20 mM LY294002, SH-5 or L-NAME under the HSS condition, respectively. As shown in Fig. 4, the inhibitions by LY294002, SH-5 or L-NAME resulted in promoted cell viability (Fig. 4A) and decreased cell apoptosis (Figs. 4B and C), decreased ROS (Fig. 4D) and NO (Fig. 4E) productions, decreased IL-1β, IL-6, MCP-1 and TNF-α mRNA and protein levels (Figs. 4F and G), and increased cell migration (Fig. 4H). Since the ABCA1 overexpression inhibited the PI3K/Akt/eNOS axis as indicated above, we suggested that ABCA1 might regulate the PI3K/Akt/eNOS pathway under HSS conditions to modulate the phenotypes of HBMECs.

## Discussion

In this study, we found that, compared to the static condition, HSS inhibited the viability and migration of HBMECs, induced apoptosis, inflammatory cytokines levels, ROS production as well as NO production, and inhibited ABCA1 expression while activating the PI3K/Akt/eNOS pathway. In addition, the overexpression of ABCA1 reversed the effect of HSS on HBMECs while contrary results were recorded with ABCA1 silencing. In addition, ABCA1 was found as an inhibitor of PI3K/Akt/eNOS pathway under HSS conditions. Furthermore, the inhibition of PI3K/Akt/eNOS pathway mimicked the effect of ABCA1 overexpression on HBMECs, which was opposite to the effects of HSS. These results suggested that ABCA1 overexpression exerts protective role against HSS-induced injury of HBMECs via regulating PI3K/Akt/eNOS signaling.

The effect of HSS on BMECs has been studied previously; it was reported that HSS corrects ischemia/reperfusion-induced neuronal dysfunction by upregulating the PECAM-1-eNOS-NO pathway [10], suggesting that HSS exerts protective role on injury BMECs. However, the effect of different profiles of SS on HBMECs has not been well elucidated so far, and the underlying mechanism still needs to be elucidated. In this study, HSS was compared to static and PSS conditions; the results indicated that HSS induces the injury of HBMECs and, in general, no significant difference was found between the PSS and the static conditions. In the present study, we initially focused on the alteration of ABCA1 in response to different SS conditions. We observed a decreased level of ABCA1 in HBMECS loaded with HSS, which was supported by a previous report showing that prolonged exposure of LSS lead to significantly elevated ABCA1 expression in HUBVC (human umbilical vein endothelial cells) as compared to HSS treatment [36]. Down-regulation of ABCA1 in HSS loaded HBMECS might hamper the cholesterol efflux, which was consistent with the deleterious effect of HSS on HBMECs. This was corroborated with the inhibitory effect of ABCA1 on the cholesterol metabolism-related genes MMP9, AQP4, and CYP46. This finding was consistent with our previous findings suggesting that cholesterol efflux is impaired by HSS [50].

Our results also showed the activation of PI3K/Akt/eNOS pathway following HSS, which was supported by previous research works [28]. Elevated endothelial migration and reduced apoptosis are crucial for maintaining endothelium integrity; our results highlighted the deleterious effect of HSS in cerebral-endothelial cells, which is consistent with previous reports [37, 40]. In addition, since CYP46 could handle surplus cholesterol that induces apoptosis [48], we speculated that the elevated CYP46 might also contribute to the reduced apoptosis in HSS group. In addition, we found that HSS inhibited the migration of HBMECs; the HSS-induced inhibition of cell migration was in corroboration with a previous research which disclosed that HSS inhibits the proliferation and migration of endothelial cells in a coculture system with VSMCs while increasing apoptotic cell death by inhibiting the p38MAPK and ERK1/2 pathways [51]. However, this observation was contradictory to a previous study indicating that LSS causes inhibition of the migration of bovine aortic endothelial cell as compared to static condition while HSS increases the speed of migration of these cells [52]. Similarly, reports have also indicated that HSS increases the migration speed and directional migration of neuroblastoma IMR32 cells via regulating the MYCN protein [53]. Thus, it seems that the effect of HSS on cell migration may be controversial and may be depends on cell types, cell origin and the level of SS applied. Further studies are needed to elucidate the correlation between HBMECs and HSS.

To further confirm the possible correlation between ABCA1 and PI3K/Akt/eNOS pathway, we performed overexpression and knockdown of ABCA1 in HBMECs, respectively. As a result, significant mRNA-level changes of ABCA1 were observed, indicating successful transfection; however, alterations concerning PI3K/Akt/eNOS at mRNA level were barely observed. Nevertheless, at protein level, HBMECs with overexpressed ABCA1 displayed significantly decreased phosphorylation level of PI3K/Akt/eNOS pathway, while the results of their ABCA1-knockdown counterparts were quite the opposite. These results suggested that the regulatory role of ABCA1 might converge on the activation of PI3K/Akt/eNOS pathway. Considering that nitric oxide (NO), an end product of PI3K/Akt/eNOS pathway, plays a fundamental role in maintaining the endothelial function, and associated with cholesterol level, we suggested that under HSS condition, alteration in NO concentration might explain the regulatory effects of ABCA1-PI3K/Akt/eNOS axis.

To confirm the regulatory role of PI3K/Akt/eNOS pathway, PI3K inhibitor (LY294002), AKT inhibitor (SH-5) and eNOS inhibitor (L-NAME) were used to treat HBMECS under HSS conditions, respectively. These inhibitors reversed the effects of HSS on different processes of HBMECs. These observations were supported by previous report [54] and highlighted the role of PI3K/Akt/eNOS pathway on cerebral-endothelial survival under HSS. In addition, since ABCA1 inhibited PI3K/Akt/eNOS pathway and because similar results were recorded for ABCA1 overexpression and the PI3K/Akt/eNOS pathway inhibitors, we stipulated that the effect of HSS on HBMECs was driven by ABCA1 modulation of the PI3K/Akt/eNOS pathway.

Although we have achieved significant results in current study, there are still some points that we would like to address in the future. For example, to further investigate and verify the interaction between ABCA1 and PI3K/Akt/eNOS pathway, especially focusing on the underlying mechanism through which ABCA1 promotes the phosphorylation, as well as measuring the change in cholesterol level caused by altered ABCA1.

In conclusion, ABCA1 has emerged as a potential mediator in HBMECS exposed to HSS, whose regulatory role could be explained by activation of several potential down-stream markers along with activation of PI3K/Akt/eNOS pathway. The potential regulatory cascade involving ABCA1 and PI3K/Akt/eNOS pathway might shed a light on the cerebral-protective role of HSS.

## Materials and methods

### Reagents

Human brain microvascular endothelial cells (HBMECs) were bought from American Type Culture Collection (ATCC CRL-3245). LY294002, N^G^-Nitro-L-Arginine Methyl Ester (L-NAME) and Annexin V-FITC fluorescein apoptosis detection kit was purchased from Sigma (St. Louis, USA). Caspase-Glo 3/7 Assay Kit was purchased from Promega (Madison, USA). SH-5 was purchased from abcam (Burlingame, USA). RNeasy Mini kit was purchased from Qiagen (Dusseldorf, Germany). PrimeScript RT reagent Kit was purchased from Takara (Dalian, China). Anti-mouse and anti-rabbit secondary antibody were purchased from Invitrogen (Carlsbad, USA). Primary antibodies against ABCA1 (sc-58219), AQP4 (sc-32739), CYP46 (sc-136148), MMP-9 (sc-21733) were purchased from Santa Cruz (CA, USA). Antibodies against PI 3 Kinase p85 (ab86714), AKT (ab8805) and eNOS (ab76198) were purchased from Abcam (Cambridge, UK). Antibodies against phospho-PI3 Kinase p85 (Tyr458)/p55(Tyr199) (CST4228), phospho-Akt (Ser473) (CST4060) and phospho-eNOS (Ser1177) (CST9570) were purchased from Cell Signaling Technology (Waltham, USA). siRNA targeting ABCA1, and corresponding negative controls were purchased from Sangon Biotech (Shanghai, China). ELISA kits for TNF-α, IL-1β, IL-6 and MCP-1 were from R&D (Minneapolis, MN).

### Knockdown and overexpression of ABCA1

Small interfering RNA (siRNA) targeting ABCA1 was used to transiently suppress ABCA1 mRNA/protein expression, and nontargeting siRNA was used as scrambled control. Briefly, total RNA of HBMECs was extracted and reverse-transcribed; the resulting cDNA was used as template for amplifying ABCA1 coding sequencing (CDS) region. The amplified ABCA1 sequence was cloned into the pcDNA-3.0 vector for plasmid recombination prior to packaging. siRNA and recombinant pcDNA 3.0-ABCA1 vector, as well as the corresponding controls were transfected into HBMECs using lipofectamine 2000, respectively. The successfully constructed ABCA1 overexpression HBMECs were named as ABCA1-vector, as compared to its control counterparts which were termed Ctrl-vector. HBMECs transfected with scramble control siRNA or ABCA1-inhibitory siRNA were abbreviated as siNC and ABCA1-siRNA, respectively.

### Cell culture, treatments and shear stress application

Low-passage (< passage 5) human brain vascular endothelial cells (HBMECs) were cultured on endothelium cell basal medium supplemented with growth factors, 1% FBS, penicillin–streptomycin. SS of 15 dyn/cm^2^ has been used in an in vitro brain endothelial cell model to mimic in vivo environment [55]; other studies have also indicated that PSS ranges between 10 and 25 dyn/cm^2^ while SS of < 5 dyn/cm^2^ is considered LSS [56–58]. Therefore, SS that is greater than 25 dyn/cm^2^ is defined as HSS; in a previous study, a SS of 40 dyn/cm^2^ was applied to HBMECs as HSS in static flow conditions [12]. Therefore, in this study, SS of 40 dyne/cm^2^ was applied as HSS while SS of 15 dyne/cm^2^ was applied as PSS. The protocol was in accordance with a protocol described previously [59]. The HBMECs grown in the absence of flow (0 dyne/cm^2^) in static conditions were used as control. For SS application, we employed a parallel plated flow chamber system according to a previous report (60), whereby we imposed a controlled level of HSS or PSS on cells that had reached confluency. The foregoing treatments were sustained for 72 h, whereas cells in control group were cultured under static conditions for the same duration. After SS loading, each group of cells were harvested and subjected to subsequent experiments. To explore the involvement of ABCA1 in the functional effect of HSS, HBMECs transfected with ABCA1-vector, ABCA1-siRNA or their respective controls were subjected to HSS loading. Moreover, to further verify the potential role of PI3K/Akt/eNOS pathway under HSS, cells were divided into 4 groups; the control group (treated with dimethyl sulfoxide (DMSO)) and 3 inhibitor groups treated respectively with one of the following inhibitors: PI3K inhibitor (LY294002) or AKT inhibitor (SH-5) or eNOS inhibitor (L-NAME). The inhibitors were dissolved in DMSO. The four groups were then subjected to HSS load as indicated above.

### RNA extraction and qRT-PCR

RNeasy Mini kit was used to extract and purify RNA from HBMECS with or without indicated treatments. Measurement of concentration and purity of RNA were performed in NanoDrop 2000 spectrometer (Thermo Fisher Scientific), the qualified RNA samples were subjected to subsequent reverse-transcription using PrimeScript RT reagent Kit. Afterwards, the qPCR reactions were carried out using the following conditions: one cycle for pre-denaturation at 95 °C for 20 s, one cycle for annealing at 58 °C for 30 s, and 40 cycles for extension at 72 °C for 30 s. qPCR reactions were repeated in triplicate. The relative Ct value was normalized against internal control β-actin, followed by 2^−ΔΔCt^ method for calculating relative expression. Primer pairs used in current study were shown in Table 1.

**Table 1:** Primer sequences

Gene	Sequence
ABCA1-F	5′-AGGCTTGTCAAGGGGTAGGA-3′
ABCA1-R	5′- GCAGCAGCTGACATGTTTGT-3′
MMP-9-F	5′- TCTATGGTCCTCGCCCTGAA-3′
MMP-9-R	5′- TTGTATCCGGCAAACTGGCT-3′
AQP4-F	5′- CCCCTAACACTCCAAAAACCCA-3′
AQP4-R	5′- CTGCAGGTCCAAAGGATCGG-3′
CYP46-F	5′- CCTGAGTCGGTTAAGAAGTTCC-3′
CYP46-R	5′- TAGGTGCTGAACAAGAGCGG-3′
PIK3-F	5′- AGGCTTGTCAAGGGGTAGGA-3′
PIK3-R	5′- GCAGCAGCTGACATGTTTGT-3′
Akt-F	5′- GAAGACGGGAGCAGGCG -3′
Akt-R	5′- AAGGTGCGTTCGATGACAGT -3′
eNOS-F	5′- GACCCACTGGTGTCCTCTTG -3′
eNOS-R	5′- CTCCGTTTGGGGCTGAAGAT -3′
β-actin-F	5′- CACCATTGGCAATGAGCGGTTC-3′
β-actin-R	5′- AGGTCTTTGCGGATGTCCACGT -3′
IL-1β-F	5′-AGCCATCATTTCACTGGCGA -3′
IL-1β-R	5′-GTAGCCGTCATGGGGAAGTC -3′
IL-6-F	5′-GCTTCCCTCAGGATGCTTGT -3′
IL-6-R	5′-ATTAACTGGGGTGCCTGCTC -3′
MCP-1-F	5′-ATGGACCATCCAAGCAGACG-3′
MCP-1-R	5′-CCCTTGCTCCACAAGGAAGA-3′
TNF-α-F	5′- GAGACAGATGTGGGGTGTGAG -3′
TNF-α-R	5′- TCCTAGCCCTCCAAGTTCCA -3′

### Western blot analysis

Cells were harvested for protein extraction. PBS buffer was used for removing the residual medium in culture flask. The rinsing was repeated three times prior to the addition of lysis buffer in conjunction with protease inhibitor cocktail. The extracted total protein of each sample was quantitated using BCA kit (Thermo Fisher Scientific) in compliance with the manufacture’s protocol. Lysate samples containing equivalent amounts of protein were mixed with loading buffer and subjected to boiling water bath for 10 min, followed by electrophoresis with SDS-PAGE gels. The separated proteins were electrically transferred to PVDF membrane, blocked and underwent incubation with primary antibodies at 4 °C overnight. The next day, the membrane was washed three time with TBST prior to incubation with secondary antibody for 1 h at ambient temperature. Finally, the secondary antibodies were removed through rinsing with TBST for three times, followed by visualization with BeyoECL Star Kit (Beyotime Biotechnology) in ECL™ Western Blotting System (GE Healthcare). The determine the relative intensities, gray value of each protein/phosphoprotein band was normalized to that of internal control β-actin or corresponding total protein (e.g. P-PI3K *versus* PI3K) using image J.

### Cell migration analysis

Cell migration was evaluated using transwell assay. Cells were adjusted to 5 × 10^5^ cells/ml with serum free medium; 100 μl of cell suspensions were seeded onto the upper chamber while the lower well was supplemented with 500 μl of culture medium containing 10% FBS. The incubation lasted 24 h at 37 °C in 5% CO_2_, the remaining cells on the upper level were gently scraped off, while cells that migrated to the lower side were fixed with methanol for 10 min and stained with 0.1% crystal violet solution. The migrated cells in three randomly selected fields were photographed at 100×magnification and counted. The cell migration rates were calculated with the following formula: cell migration rate = number of migrated cells in experiment groups/number of migrated cells in control group × 100%.

### Flow cytometry analysis

The apoptosis of treated HBMECS was evaluated through flow cytometry. Approximate 1 × 10^6^ HBMECS were collected, washed three times with pre-cooled PBS buffer prior to centrifugation at 1000 g for 5 min. Afterwards, the precipitated cells were incubated with 500 μL 1 × Annexin binding buffer. Subsequently, each cell suspension was mixed with 5 μL Annexin-V-FITC and 10 μL propidium iodide (PI), incubated at room temperature for 15 min and protected from light. Cell apoptosis was quantified using the total population of Annexin V-FITC-positive cells. Analysis of cell apoptosis was carried out in FACS Calibur (BD Biosciences), the resultant data was analyzed using Flow Jo.

### Cell survival assay

The cell apoptosis results from flow cytometry were complemented by caspase 3/7 assays. Briefly, HBMECS were pre-seeded onto a 96-well plate at a density of 1 × 10^5^ cells/well overnight. After cell adherence, 50 μl of caspase Glo 3/7 reagent was added into each well, the reaction was left to incubation for 2 h at RT by constant shaking. The resultant luminescence produced by cleaved caspase was evaluated using a GloMax 96 Microplate luminometer (Promega).

### ELISA detection of cytokines

The levels of IL-1β, IL-6, MCP-1 and TNF-α in the supernatant was measured using the specific ELISA kits according to the protocols indicated by the vendors.

### Caspase 3/7 activity

The caspase 3/7 activity of cells was detected using the Cell Meter™ Caspase 3/7 Activity Apoptosis Assay Kit according to the manufacturer’s instructions.

### Determination of reactive oxygen species (ROS) production

We first prepared fresh stock solution of carboxy-H2DCFDA in sterile dimethylsulfoxide (DMSO) before the experiment. The cells were washed with phosphate-buffered saline (PBS) to remove traces of the original medium. The cells were loaded with the dye and the control dye. The cells were then incubated in a conventional incubator (37 °C, 5% CO_2_) in the dark for 30 min, and discarded all unstained dye solutions. The medium containing carboxy-H2DCFDA was removed and the cells were washed twice with PBS. Subsequently, we added fresh medium without any supplements to the carboxy-H2DCFDA-loaded cells and incubate as required. Finally, we detected ROS by immediately analyzing the cells by flow cytometry using the FL1 channel.

### Determination of nitric oxide (NO) level

Before the experiment, we first prepared N-1-napthylethylenediamine dihydrochloride (NED), Sulfanilamide and Nitrite standard solutions. Briefly, we cultured cells in 96-well plates. We used lipopolysaccharide (LPS) (100 ng/ml) and recombinant IL-4 to treat cells to induce NO production. After that, we spined and collected supernatants and transferred 50 μl to a new 96-well plate. We added 50 μl of Sulfanilamide Solution to each sample and mix well. The cells were incubated at room temperature for 10 min in the dark. We added 50 μl of N-1-napthylethylenediamine dihydrochloride (NED) solution to each sample, controlled well and mix well. The cells were incubated at room temperature for 10 min in the dark. Finally, the cells were measured absorbance immediately using a plate reader with filter of wavelengths between 520 and 550 nm.

### Statistical analysis

Data obtained from at least three independent experiments (different passages of HBMEC) are presented as mean ± SEM. Statistical analyses were performed using Graphpad Prism software. Statistical significance between two groups were determined using a two-tailed t-test, and those among more than two groups were analyzed by one-way analysis of variance (ANOVA). Results with *p* < 0.05 were considered statistically significant.

## Supplementary Information

**Additional file 1: Figure S1.** Full-length blots/gels of ABCA1, MMP9, AQP4, CYP46, and beta-actin are presented. **Figure S2.** Full-length blots/gels of phospho-PI3K, PI3K, AKT, phospho-AKT, eNOS, phospho-eNOS and beta-actin are presented. **Figure S3.** Full-length blots/gels of ABCA1 and beta-actin are presented. **Figure S4.** Full-length blots/gels of MMP9, AQP4, CYP46 and β-actin are presented. **Figure S5.** Full-length blots/gels of phospho-PI3K, PI3K, AKT, phospho-AKT, eNOS, phospho-eNOS and β-actin are presented.

## Data Availability

All data generated or analyzed during this study are included in this published article and source data are available from the corresponding upon reasonable request.

## Abbreviations

SS: Shear stress
ECs: Endothelial cells
HBMECs: Human brain microvascular endothelial cells
ABCA1: ATP-binding cassette transporter A1
PI3Ks: Phosphoinositide 3-kinases
eNOS: Endothelial NO synthase
siRNA: Small interfering RNA

## References

[1] Wang X, Xu B, Xiang M, Yang X, Liu Y, Liu X (2020). Advances on fluid shear stress regulating blood-brain barrier. Microvasc Res.

[2] Godinho-Pereira J, Garcia AR, Figueira I, Malhó R, Brito MA (2021). Behind brain metastases formation: cellular and molecular alterations and blood-brain barrier disruption. Int J Mol Sci..

[3] Noorani B, Bhalerao A, Raut S, Nozohouri E, Bickel U, Cucullo L (2021). A quasi-physiological microfluidic blood-brain barrier model for brain permeability studies. Pharmaceutics..

[4] Wang YI, Abaci HE, Shuler ML (2017). Microfluidic blood-brain barrier model provides in vivo-like barrier properties for drug permeability screening. Biotechnol Bioeng.

[5] Tian S, Bai Y, Yang L, Wang X, Wu Y, Jia J (2013). Shear stress inhibits apoptosis of ischemic brain microvascular endothelial cells. Int J Mol Sci.

[6] Ando J, Yamamoto K (2009). Vascular mechanobiology: endothelial cell responses to fluid shear stress. Circ J.

[7] Guo FX, Hu YW, Zheng L, Wang Q (2017). Shear stress in autophagy and its possible mechanisms in the process of atherosclerosis. DNA Cell Biol.

[8] Chien S, Li S, Shyy YJ (1998). Effects of mechanical forces on signal transduction and gene expression in endothelial cells. Hypertension.

[9] Rundek T, Della-Morte D (2015). The role of shear stress and arteriogenesis in maintaining vascular homeostasis and preventing cerebral atherosclerosis. Brain Circu..

[10] Gao JQ, Wang P, Yan JW, Ba LN, Shi PL, Wu HM (2020). Shear stress rescued the neuronal impairment induced by global cerebral ischemia reperfusion via activating PECAM-1-eNOS-NO Pathway. Front Cell Dev Biol.

[11] DeStefano JG, Xu ZS, Williams AJ, Yimam N, Searson PC (2017). Effect of shear stress on iPSC-derived human brain microvascular endothelial cells (dhBMECs). Fluids Barriers CNS.

[12] Garcia-Polite F, Martorell J, Del Rey-Puech P, Melgar-Lesmes P, O'Brien CC, Roquer J (2017). Pulsatility and high shear stress deteriorate barrier phenotype in brain microvascular endothelium. J Cerebral Blood Flow Metab : official journal of the International Society of Cerebral Blood Flow and Metabolism.

[13] Sene A, Khan AA, Cox D, Nakamura RE, Santeford A, Kim BM (2013). Impaired cholesterol efflux in senescent macrophages promotes age-related macular degeneration. Cell Metab.

[14] Liang Z, Li W, Yang S, Liu Z, Sun X, Gao X (2018). Tangier disease may cause early onset of atherosclerotic cerebral infarction: a case report. Medicine (Baltimore).

[15] Glass CK, Saijo K, Winner B, Marchetto MC, Gage FH (2010). Mechanisms underlying inflammation in neurodegeneration. Cell.

[16] Schain M, Kreisl WC (2017). Neuroinflammation in neurodegenerative disorders—a review. Curr Neurol Neurosci Rep.

[17] Stephenson J, Nutma E, van der Valk P, Amor S (2018). Inflammation in CNS neurodegenerative diseases. Immunology.

[18] Subhramanyam CS, Wang C, Hu Q, Dheen ST (2019). Microglia-mediated neuroinflammation in neurodegenerative diseases. Semin Cell Dev Biol.

[19] Vejux A (2021). Cell Death, inflammation and oxidative stress in neurodegenerative diseases: mechanisms and cytoprotective molecules. Int J Mol Sci..

[20] Dietrich JB (2002). The adhesion molecule ICAM-1 and its regulation in relation with the blood-brain barrier. J Neuroimmunol.

[21] Mantle JL, Lee KH (2018). A differentiating neural stem cell-derived astrocytic population mitigates the inflammatory effects of TNF-α and IL-6 in an iPSC-based blood-brain barrier model. Neurobiol Dis.

[22] Al-Obaidi MMJ, Desa MNM (2018). Mechanisms of blood brain barrier disruption by different types of bacteria, and bacterial-host interactions facilitate the bacterial pathogen invading the brain. Cell Mol Neurobiol.

[23] Hsu CP, Lin CH, Kuo CY (2018). Endothelial-cell inflammation and damage by reactive oxygen species are prevented by propofol via ABCA1-mediated cholesterol efflux. Int J Med Sci.

[24] Lu J, Chen X, Xu X, Liu J, Zhang Z, Wang M (2019). Active polypeptides from Hirudo inhibit endothelial cell inflammation and macrophage foam cell formation by regulating the LOX-1/LXR-α/ABCA1 pathway. Biomed Pharmacother Biomed Pharmacother..

[25] Stamatikos A, Dronadula N, Ng P, Palmer D, Knight E, Wacker BK (2019). ABCA1 Overexpression in endothelial cells in vitro enhances ApoAI-mediated cholesterol efflux and decreases inflammation. Hum Gene Ther.

[26] Huang K, Jo H, Echesabal-Chen J, Stamatikos A (2021). Combined LXR and RXR agonist therapy increases ABCA1 protein expression and enhances apoai-mediated cholesterol efflux in cultured endothelial cells. Metabolites..

[27] Xie Q, Peng J, Guo Y, Li F (2021). MicroRNA-33-5p inhibits cholesterol efflux in vascular endothelial cells by regulating citrate synthase and ATP-binding cassette transporter A1. BMC Cardiovasc Disord.

[28] Chen Z, Li T, Kareem K, Tran D, Griffith BP, Wu ZJ (2019). The role of PI3K/Akt signaling pathway in non-physiological shear stress-induced platelet activation. Artif Organs.

[29] Shi X, Wang J, Lei Y, Cong C, Tan D, Zhou X (2019). Research progress on the PI3K/AKT signaling pathway in gynecological cancer (Review). Mol Med Rep.

[30] Balligand JL, Feron O, Dessy C (2009). eNOS activation by physical forces: from short-term regulation of contraction to chronic remodeling of cardiovascular tissues. Physiol Rev.

[31] Duran WN, Breslin JW, Sanchez FA (2010). The NO cascade, eNOS location, and microvascular permeability. Cardiovasc Res.

[32] Schini VB (1996). The L-Arginine-Nitric oxide pathway in the vascular smooth muscle: regulation and pathophysiological significance. Vasc Endothelium Responses Injury..

[33] Dimmeler S, Zeiher AM (1999). Nitric oxide-an endothelial cell survival factor. Cell Death Differ.

[34] Roviezzo F, Cuzzocrea S, Di Lorenzo A, Brancaleone V, Mazzon E, Di Paola R (2007). Protective role of PI3-kinase-Akt-eNOS signalling pathway in intestinal injury associated with splanchnic artery occlusion shock. Br J Pharmacol.

[35] Andrews AM, Muzorewa TT, Zaccheo KA, Buerk DG, Jaron D, Barbee KA (2017). Cholesterol enrichment impairs capacitative calcium entry, eNOS phosphorylation & shear stress-induced NO production. Cell Mol Bioeng.

[36] Vion AC, Ramkhelawon B, Loyer X, Chironi G, Devue C, Loirand G (2013). Shear stress regulates endothelial microparticle release. Circ Res.

[37] Paone S, Baxter AA, Hulett MD, Poon IKH (2019). Endothelial cell apoptosis and the role of endothelial cell-derived extracellular vesicles in the progression of atherosclerosis. Cell Mol Life Sci.

[38] Zhang T, Tian F, Wang J, Jing J, Zhou SS, Chen YD (2015). Atherosclerosis-associated endothelial cell apoptosis by MiR-429-Mediated down regulation of Bcl-2. Cell Physiol Biochem.

[39] Shiu Y-T, Li S, Marganski WA, Usami S, Schwartz MA, Wang Y-L (2004). Rho mediates the shear-enhancement of endothelial cell migration and traction force generation. Biophys J.

[40] Yang Y, Jamilpour N, Yao B, Dean ZS, Riahi R, Wong PK (2016). Probing leader cells in endothelial collective migration by plasma lithography geometric confinement. Sci Rep.

[41] Salo T, Makela M, Kylmaniemi M, Autio-Harmainen H, Larjava H (1994). Expression of matrix metalloproteinase-2 and -9 during early human wound healing. Lab Invest.

[42] Yang Y, Rosenberg GA (2015). Matrix metalloproteinases as therapeutic targets for stroke. Brain Res.

[43] Pijet B, Stefaniuk M, Kaczmarek L (2019). MMP-9 contributes to dendritic spine remodeling following traumatic brain injury. Neural Plast.

[44] Todd N, Zhang Y, Xu K, Liu Y, Erokwu BO, Zhao P (2019). Increased cerebral vascularization and decreased water exchange across the blood-brain barrier in aquaporin-4 knockout mice. Plos ONE..

[45] Katoozi S, Skauli N, Zahl S, Deshpande T, Ezan P, Palazzo C (2020). Uncoupling of the astrocyte syncytium differentially affects AQP4 isoforms. Cells..

[46] Chu H, Xiang J, Wu P, Su J, Ding H, Tang Y (2014). The role of aquaporin 4 in apoptosis after intracerebral hemorrhage. J Neuroinflammation.

[47] Martin MG, Trovo L, Perga S, Sadowska A, Rasola A, Chiara F (2011). Cyp46-mediated cholesterol loss promotes survival in stressed hippocampal neurons. Neurobiol Aging.

[48] Huang YN, Lin CI, Liao H, Liu CY, Chen YH, Chiu WC (2016). Cholesterol overload induces apoptosis in SH-SY5Y human neuroblastoma cells through the up regulation of flotillin-2 in the lipid raft and the activation of BDNF/Trkb signaling. Neuroscience.

[49] Bernas MJ, Cardoso FL, Daley SK, Weinand ME, Campos AR, Ferreira AJ (2010). Establishment of primary cultures of human brain microvascular endothelial cells to provide an in vitro cellular model of the blood-brain barrier. Nat Protoc.

[50] Li Z, Li JN, Li Q, Liu C, Zhou LH, Zhang Q (2021). miR-25-5p regulates endothelial progenitor cell differentiation in response to shear stress through targeting ABCA1. Cell Biol Int.

[51] Ji Q, Wang YL, Xia LM, Yang Y, Wang CS, Mei YQ (2019). High shear stress suppresses proliferation and migration but promotes apoptosis of endothelial cells co-cultured with vascular smooth muscle cells via down-regulating MAPK pathway. J Cardiothorac Surg.

[52] Gojova A, Barakat AI (2005). Vascular endothelial wound closure under shear stress: role of membrane fluidity and flow-sensitive ion channels. J Appl Physiol (Bethesda, Md : 1985)..

[53] Hiraiwa T, Yamada TG, Miki N, Funahashi A, Hiroi N (2019). Activation of cell migration via morphological changes in focal adhesions depends on shear stress in MYCN-amplified neuroblastoma cells. J R Soc Interface.

[54] Wojciak-Stothard B, Ridley AJ (2003). Shear stress-induced endothelial cell polarization is mediated by Rho and Rac but not Cdc42 or PI 3-kinases. J Cell Biol.

[55] Booth R, Kim H (2014). Permeability analysis of neuroactive drugs through a dynamic microfluidic in vitro blood-brain barrier model. Ann Biomed Eng.

[56] Rabinovitch M, Konstam MA, Gamble WJ, Papanicolaou N, Aronovitz MJ, Treves S (1983). Changes in pulmonary blood flow affect vascular response to chronic hypoxia in rats. Circ Res.

[57] Malek AM, Alper SL, Izumo S (1999). Hemodynamic shear stress and its role in atherosclerosis. JAMA.

[58] Davies PF, Spaan JA, Krams R (2005). Shear stress biology of the endothelium. Ann Biomed Eng.

[59] Mahler GJ, Frendl CM, Cao Q, Butcher JT (2014). Effects of shear stress pattern and magnitude on mesenchymal transformation and invasion of aortic valve endothelial cells. Biotechnol Bioeng.

[60] Sun J, Luo Q, Liu L, Song G (2018). Low-level shear stress promotes migration of liver cancer stem cells via the FAK-ERK1/2 signalling pathway. Cancer Lett.

