# Supplementary material for: Cholesterol efflux regulator ABCA1 exerts protective role against high shear stress-induced injury of HBMECs via regulating PI3K/Akt/eNOS signaling
Source: BMC Neurosci. 2022 Nov 5;23:61. doi: 10.1186/s12868-022-00748-2 (PMC9636808; doi:10.1186/s12868-022-00748-2)
Supplement: Supplementary file 1 — Additional file 1: Figure S1. Full-length blots/gels of ABCA1, MMP9, AQP4, CYP46, and beta-actin are presented. Figure S2. Full-length blots/gels of phospho-PI3K, PI3K, AKT, phospho-AKT, eNOS, phospho-eNOS and beta-actin are presented. Figure S3. Full-length blots/gels of ABCA1 and beta-actin are presented. Figure S4. Full-length blots/gels of MMP9, AQP4, CYP46 and β-actin are presented. Figure S5. Full-length blots/gels of phospho-PI3K, PI3K, AKT, phospho-AKT, eNOS, phospho-eNOS and β-actin are presented. [file 12868_2022_748_MOESM1_ESM.pptx]

## Slide 1
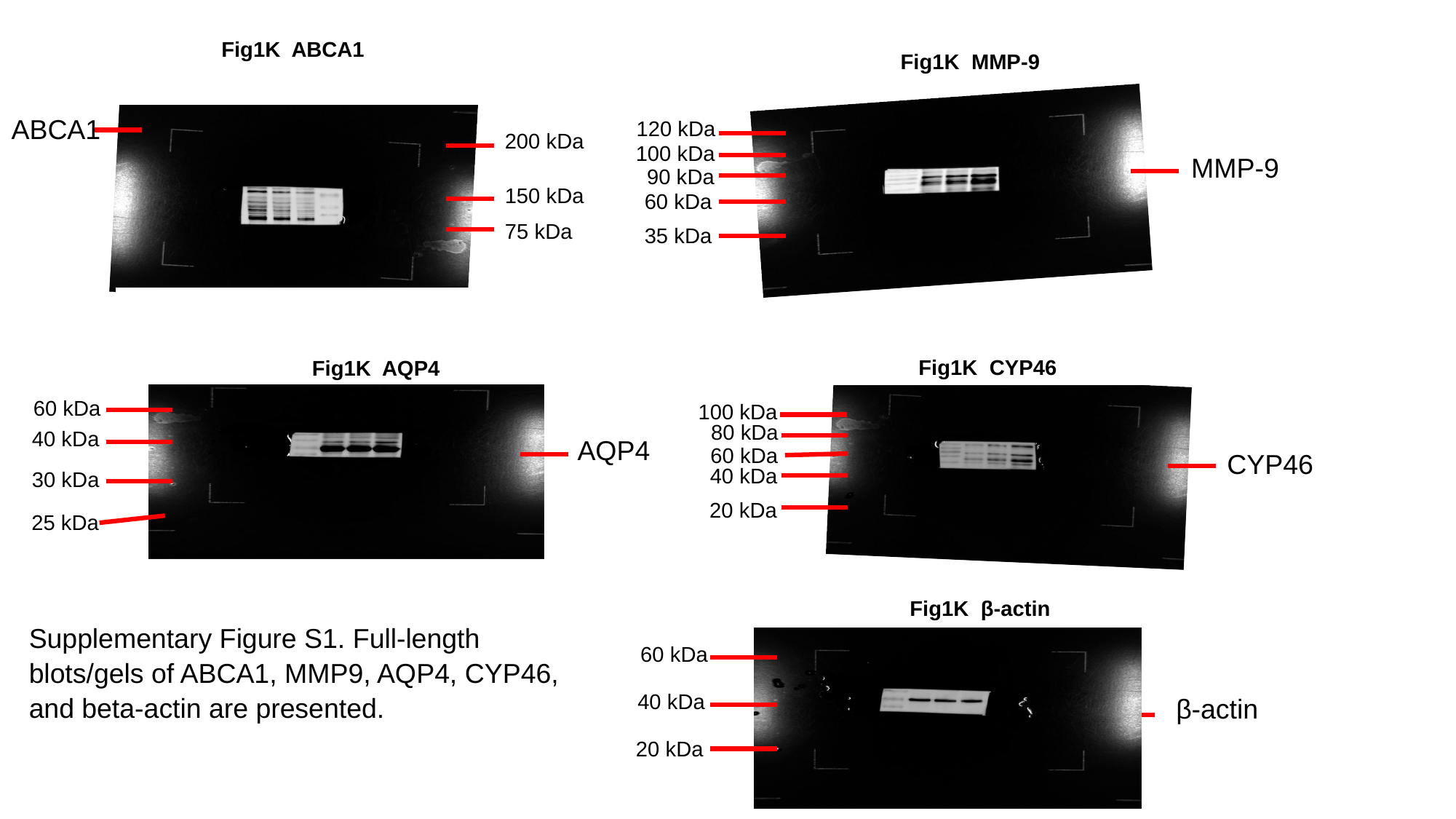

Fig1K ABCA1
200 kDa
150 kDa
75 kDa
ABCA1
Fig1K MMP-9
120 kDa
100 kDa
90 kDa
60 kDa
35 kDa
MMP-9
 Fig1K CYP46
100 kDa
80 kDa
60 kDa
40 kDa
20 kDa
CYP46
Fig1K AQP4
60 kDa
40 kDa
30 kDa
25 kDa
AQP4
Fig1K β-actin
60 kDa
40 kDa
20 kDa
β-actin
Supplementary Figure S1. Full-length blots/gels of ABCA1, MMP9, AQP4, CYP46, and beta-actin are presented.

## Slide 2
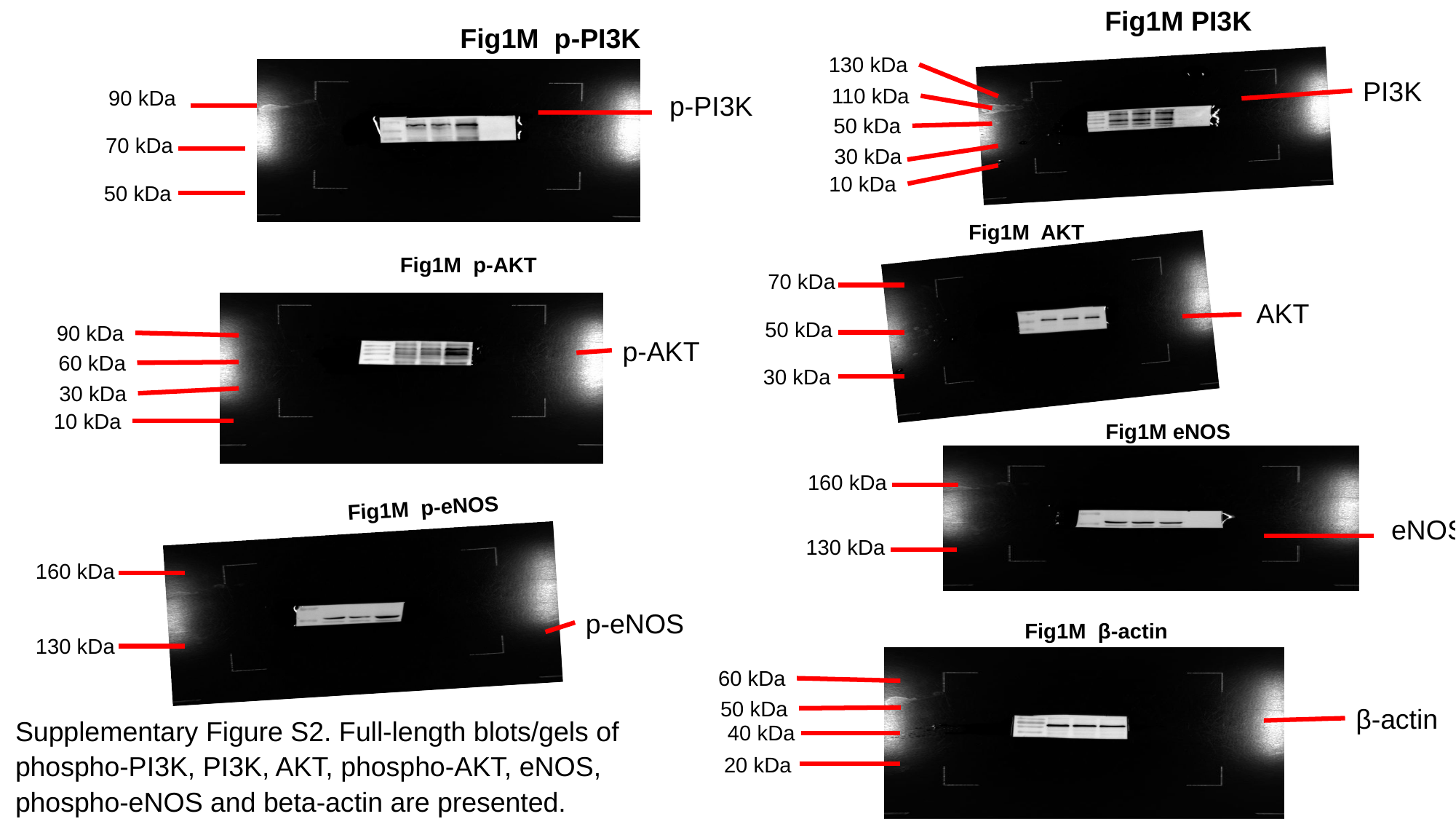

Fig1M PI3K
130 kDa
110 kDa
50 kDa
30 kDa
10 kDa
PI3K
Fig1M p-PI3K
90 kDa
70 kDa
50 kDa
p-PI3K
Fig1M AKT
70 kDa
50 kDa
30 kDa
AKT
Fig1M p-AKT
90 kDa
60 kDa
30 kDa
10 kDa
p-AKT
Fig1M eNOS
160 kDa
130 kDa
eNOS
Fig1M p-eNOS
160 kDa
130 kDa
p-eNOS
Fig1M β-actin
60 kDa
50 kDa
40 kDa
20 kDa
β-actin
Supplementary Figure S2. Full-length blots/gels of phospho-PI3K, PI3K, AKT, phospho-AKT, eNOS, phospho-eNOS and beta-actin are presented.

## Slide 3
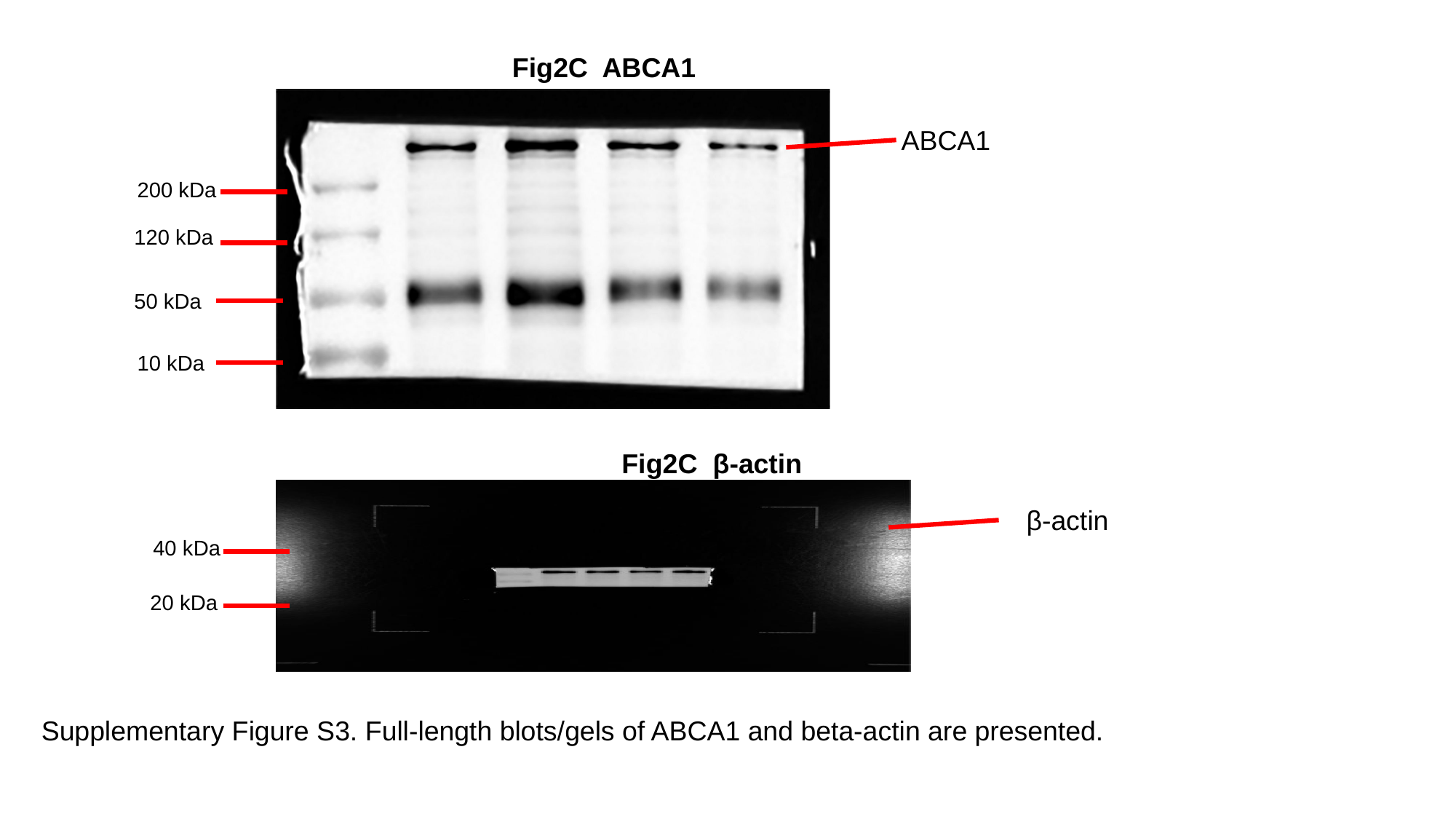

Fig2C ABCA1
ABCA1
200 kDa
120 kDa
50 kDa
10 kDa
Fig2C β-actin
β-actin
40 kDa
20 kDa
Supplementary Figure S3. Full-length blots/gels of ABCA1 and beta-actin are presented.

## Slide 4
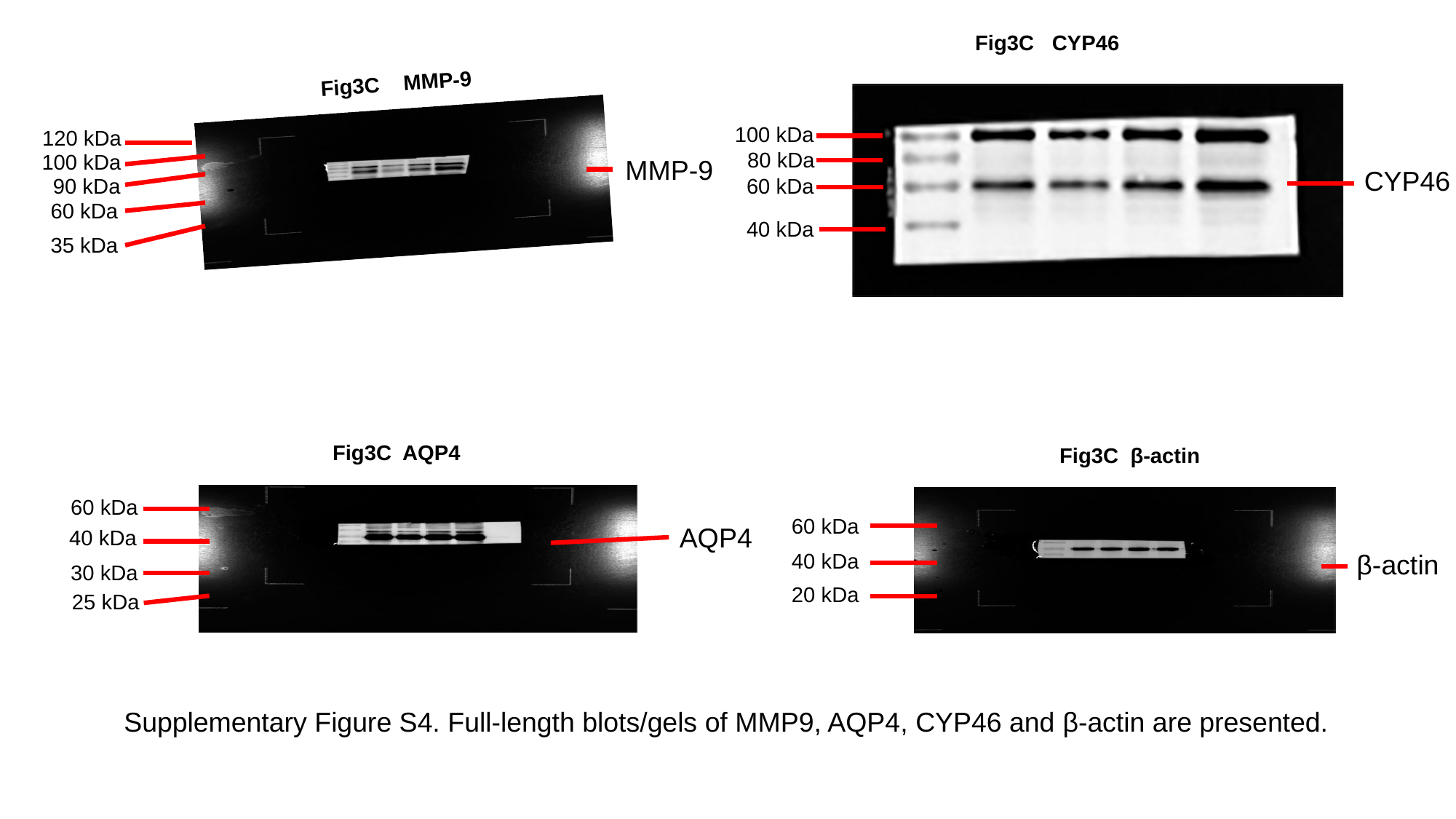

Fig3C CYP46
100 kDa
80 kDa
60 kDa
40 kDa
CYP46
Fig3C MMP-9
120 kDa
100 kDa
90 kDa
60 kDa
35 kDa
MMP-9
Fig3C AQP4
Fig3C β-actin
60 kDa
40 kDa
20 kDa
β-actin
60 kDa
40 kDa
30 kDa
25 kDa
AQP4
Supplementary Figure S4. Full-length blots/gels of MMP9, AQP4, CYP46 and β-actin are presented.

## Slide 5
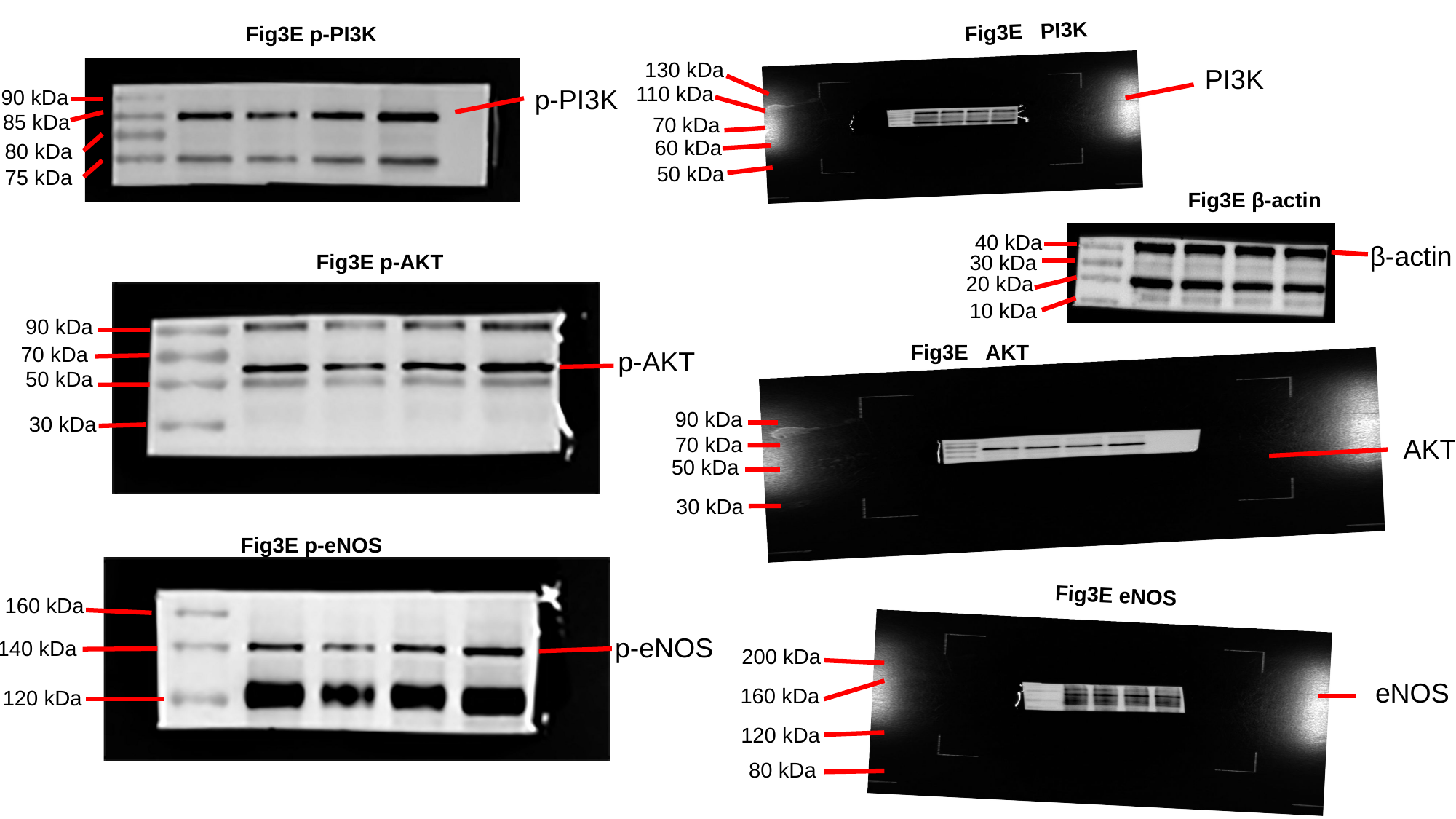

Fig3E p-PI3K
90 kDa
85 kDa
80 kDa
75 kDa
Fig3E PI3K
130 kDa
110 kDa
70 kDa
60 kDa
50 kDa
PI3K
p-PI3K
Fig3E β-actin
40 kDa
30 kDa
20 kDa
10 kDa
β-actin
Fig3E p-AKT
90 kDa
70 kDa
50 kDa
30 kDa
p-AKT
Fig3E AKT
90 kDa
70 kDa
50 kDa
30 kDa
AKT
Fig3E p-eNOS
160 kDa
140 kDa
120 kDa
p-eNOS
Fig3E eNOS
200 kDa
160 kDa
120 kDa
80 kDa
eNOS
Supplementary Figure S5. Full-length blots/gels of phospho-PI3K, PI3K, AKT, phospho-AKT, eNOS, phospho-eNOS and β-actin are presented.
